# Five newly recorded Cyprinid fish (Teleostei: Cypriniformes) in Myanmar

**DOI:** 10.24272/j.issn.2095-8137.2017.063

**Published:** 2017-09-18

**Authors:** Tao Qin, Zhi-Ying Chen, Lu-Lu Xu, Paing Zaw, Yunn Mi Mi Kyaw, Kyaw Win Maung, Xiao-Yong Chen

**Affiliations:** ^1^Southeast Asia Biodiversity Research Institute, Chinese Academy of Sciences, Yezin Nay Pyi Taw 05282, Myanmar; ^2^Kunming Institute of Zoology, Chinese Academy of Sciences, Kunming Yunnan 650223, China; ^3^Kunming Collage of Life Science, University of Chinese Academy of Sciences, Kunming Yunnan 650204, China; ^4^Forest Research Institute, Yezin Nay Pyi Taw 05282, Myanmar

**Keywords:** New record, Cyprinidae, Mali Hka River, Irrawaddy, Myanmar

## Abstract

Freshwater fish from the Putao and Myitkyina areas were collected in three ichthyofaunal surveys of the Mali Hka River and tributaries in and around Khakaborazi National Park and Hponkanrazi Wildlife Sanctuary, Kachin State, from 2014-2016. *Tor yingjiangensis* Chen et Yang 2004, *Tor qiaojiensis* Wu et al. 1977, *Garra qiaojiensis* Wu et al. 1977, *Garra bispinosa* Zhang 2005, and *Schizothorax oligolepis* Huang 1985, originally described from the upper Irrawaddy (Ayeyarwaddy) River in China, are first reported herein as new records to Myanmar. Counts, measurements, descriptions, photographs, and distributions of the specimens of the five newly recorded species are provided.

## INTRODUCTION

South and Southeast Asia are among the most speciose areas on the planet, containing 20% of all known freshwater vertebrate species and 25% of all known aquatic plants (Balian et al., 2008). There are 310 native species of freshwater fish reported in Myanmar (Davies et al., 2004), and 508 freshwater species currently present in FishBase (Froese & Pauly, 2017). The eastern Himalaya region is part of the Indo-Burma and Himalaya Biodiversity Hotspots, with at least 520 species of freshwater fish reported (Allen et al., 2010). Abell et al. (2008) presented a global map of 426 freshwater ecoregions based on the distributions and compositions of freshwater fish species. The Sittaung-Irrawaddy is one of the six freshwater ecoregions of the Eastern Himalaya assessment region, with the Irrawaddy (Ayeyarwaddy) ecoregion containing more endemic species of freshwater fish (between 119–195) than any of the other Eastern Himalayan freshwater ecosystems (Vishwanath et al., in Allen et al., 2010).

**Figure 1:**
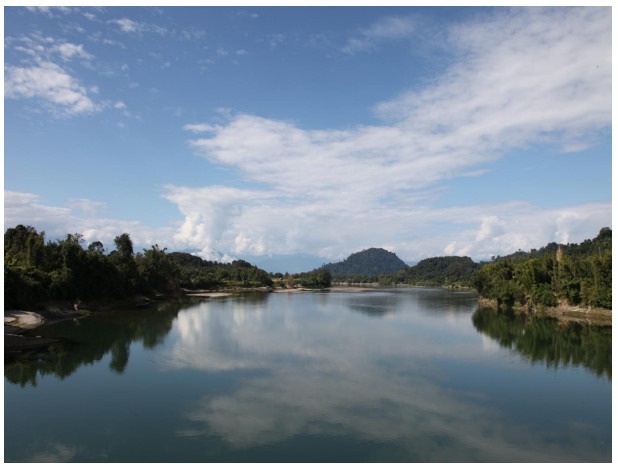
View of the main channel of Mali Hka River

Putao (Hkamti Long) is the northern most district in Myanmar, and contains two protected areas, that is, Khakaborazi National Park and Hponkanrazi Wildlife Sanctuary (Beffasti & Galanti, 2011). Twenty newly recorded species and at least three new species were reported by Chaudhuri (1919) during the earliest fish survey in the Putao area (Kachin State, northern Myanmar), with fish species from Sunprabum and Putao areas subsequently reported (Mukerji, 1933, 1934; Rendahl, 1948). Systematic study has resulted in the addition of other new species in Putao and northernmost Myanmar (Chen et al., 2017; Conway & Britz, 2010; Hora, 1921, 1923; Kottelat, 1990; Kullander, 2012; Kullander & Britz, 2002; Kullander & Fang, 2005; Ng, 2008). According to Kottelat (2015), based on a survey of the Mali Hka River (Figure 1) conducted by the Fauna & Flora and Zoology Departments of Myitkyina University from November to December 2014, a total of 42 species were collected, of which 16 species were first recorded within the Putao area, including three new records to Myanmar: *Oreinus* cf. *meridionalis* (=*Schizothorax meridionalis*), *Garra* aff. *dulongensis* (=*Placocheilus dulongensis*), and *Pseudecheneis brachyurus*.

The Southeast Asia Biodiversity Research Institute (SEABRI), Chinese Academy of Sciences (CAS), and Forest Research Institute (FRI) have organized continuous biological surveys in North Myanmar since 2014. After further study in the laboratory, identification of samples was confirmed by comparison with materials in the Kunming Natural History Museum of Zoology, Kunming Institute of Zoology (KIZ), CAS, Kunming, Yunnan, China. A new sisorid catfish species, *Oreoglanis hponkanensis* was identified and published (Chen et al., 2017), and five newly recorded species to Myanmar are reported herein, including *Tor yingjiangensis*, *Tor qiaojiensis*, *Garra qiaojiensis*, *Garra bispinosa*, and *Schizothorax oligolepis*.

## MATERIALS AND METHODS

Fish were collected by gillnet, dipnet, and electric shocker depending on the conditions of different survey sites, or were purchased from the Putao and Myitkyina fish markets, and fixed in 10% formalin. Counts and measurements were taken on the left side of the specimen whenever possible. All morphometric measurement data were recorded point-to-point using manual dial calipers and recorded to 0.1 mm. Counts and measurements followed the original descriptions provided in the literature and local annals, viz. *Tor yingjiangensis* (Chen & Yang, 2004; Chu & Chen, 1989), *Tor qiaojiensis* (Chu & Chen, 1989; Wu, 1977), *Garra qiaojiensis* (Chu & Chen, 1989; Wu, 1977), *Garra bispinosa* (Zhang, 2005), and *Schizothorax oligolepis* (Huang, 1985). GPS coordinates were obtained from a Garmin eTrex-309 handheld device. The specimens examined in the present study were deposited in the Laboratory of Aquatic Biodiversity Research Group, SEABRI, CAS, which is based in FRI, Myanmar, and in the Kunming Natural History Museum of Zoology, KIZ, CAS, China. Abbreviations: ex., examined specimens; SL, standard length; CPL, caudal-peduncle length; HL, head length; D-LL, scales between dorsal-fin origin and lateral line; V-LL, scales between pelvic-fin origin and lateral line.

## RESULTS

### *Tor yingjiangensis* Chen & Yang, 2004

*Tor yingjiangensis* Chen & Yang, 2004: 185–191 (Yingjiang, Yunnan, China).

*Tor* (*Tor*) *putitora*: Chen & Chu, 1985: 79-86 (Yingjiang, Yunnan, China).

**Material examined**: SEABRI20140098-100, 3 ex., 70.5–81.5 mm SL, main stem of Mali Hka River, Irrawaddy Basin, Wurunga Village, Naung Mun Township, Putao District, Kachin State, Myanmar (N27°30'15.3", E97°48'48.2", 543 m a.s.l.), collected by Xiao-Yong Chen and Tao Qin, 30 November to 7 December 2014; SEABRI20160086–089, 4 ex., 98.9–174.9 mm SL, from Myitkyina fish market, Myitkyina City, Kachin State, Myanmar, collected by Xiao-Yong Chen, Tao Qin and Shu-Sen Shu, 1 August 2016; SEABRI20160176, 1 ex., 75.5 mm SL, from Putao fish market, Putao District, Kachin State, Myanmar, collected by Xiao-Yong Chen, Tao Qin, and Shu-Sen Shu, 4 August 2016.

**Comparative material examined**: Holotype: KIZ164401, 181 mm SL, paratypes: KIZ704404, KIZ764229, KIZ764235–236, 4 ex., 72.5–162 mm SL, Dayingjiang River (tributary of upper Irrawaddy River), Manyun Town, Yingjiang County, Yunnan, China; KIZ2006004189, 198.9 mm SL, Nanzhang River (tributary of Longchuanjiang River, Irrawaddy Basin), Wangzishu Town, Longchuan County, Yunnan, China.

**Figure 2:**
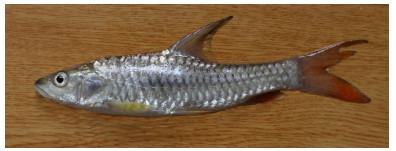
Lateral view of *Tor yingjiangensis* before preservation, SEABRI20160089, 167.4 mm SL

**Figure 3:**
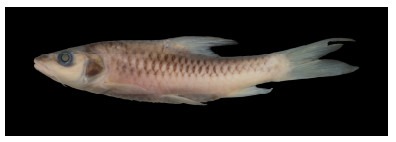
Lateral view of *Tor yingjiangensis* after preservation in formalin, SEABRI20160089, 167.4 mm SL

**Figure 4:**
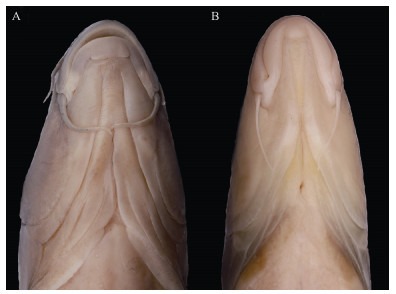
Ventral view of the head

**Description**: According to the original description of *Tor yingjiangensis* (Chen & Yang, 2004), the examined specimens can be exclusively distinguished based on the following characters: Body elongate and moderately compressed; dorsal body profile convex, slowly increasing, reaching highest point at dorsal-fin origin; ventral profile less arched; lateral head length considerably greater than body depth at dorsal-fin origin (Figures 2, 3). Snout pointed, mouth terminal, lips well fleshy, median lobe of lower lip short, posterior margin truncate, not reaching vertical across corners of mouth (Figure 4B); no tubercles on snout or sides of face; two pairs of barbels, length of rostral and maxillary barbels almost equal, longer than diameter of eye. Body entirely scaled; lateral line complete, lateral-line scales 25–26, D-LL 3.5–4, V-LL 3, circumpeduncular scales 12, no horizontal or longitudinal stripe on sides of body.

Counts and morphometric measurements of examined specimens are listed in Table 1.

**Table 1:** Comparisons of counts and proportional measurements of *Tor yingjiangensis* and *Tor qiaojiensis*

	***T*. *yingjiangensis* (*n*=8)**	***T*. *yingjiangensis*^1^(*n*=5)**	***T*. *qiaojiensis* (*n*=24)**	***T*. *qiaojiensis*^2^(*n*=9)**
Total length (mm)	90.7–226.8	82.0–238.5	72.9–234.1	57.0–292.0
Standard length (mm)	70.5–174.9	60.0–181.0	54.1–189.7	46.0–231.0
Dorsal-fin rays	ⅳ, 9	ⅳ, 9	ⅳ, 9	ⅳ, 9
Anal-fin rays	ⅲ, 5	ⅲ, 5	ⅲ, 5	ⅲ, 5
Pectoral-fin rays	ⅰ, 12–14	ⅰ, 15–16	ⅰ, 13–15	ⅰ, 15–16
Pelvic-fin rays	ⅰ, 8	ⅰ, 8–9	ⅰ, 8–9	ⅰ, 8–9
Lateral-line scales	25–26	24–26	29–30	29–30
D-LL	3.5–4	4–4.5	4.5	4.5–5.5
V-LL	3	3–3.5	2.5–3	2.5–3.5
Circumpeduncular scales	10–12	12	10	10–12
**Percentage of SL (%)**				
Body depth	24.1–28.2	25.5–27.3	24.7–28.1	27.3–31.4
Head length	28.3–33.5	28.7–33.9	26.3–31.4	27.7–30.6
Caudal-peduncle length	13.4–15.8	11.3–14.8	15.5–17.4	13.9–18.8
Caudal-peduncle depth	10.7–12.7	11.1–13.3	11.1–12.5	9.7–11.0
**Percentage of HL (%)**				
Snout length	29.1–31.8	33.3–35.4	29.7–32.2	28.4–33.7
Eye diameter	21.6–26.3	17.7–25.6	23.9–28.7	22.6–27.7
Body depth	77.0–88.3	77.1–95.2	86.7–98.5	90.4–95.5
Caudal-peduncle depth/length (%)	69.3–80.3	75.0–106.7	66.0–76.9	68.1–80.4
^1^: from Chen & Yang (2004); ^2^: from Chu & Chen (1989). Some data were converted for comparison.				

**Color in life**: Body silver, grey on dorsum. Head grey on back, silver on lateral and ventral sides. Opercule partly dark. Dorsal fin light grey, rays and distal dark grey, paired fins light yellow, caudal fin and anal fin red, but blackish distally. Scales form dark network on flank (Figure 2).

**Color in preservative**: Body and head dark grey on back, white on lateral and ventral sides. Opercule dark. All fins greyish white (Figure 3).

**Figure 7:**
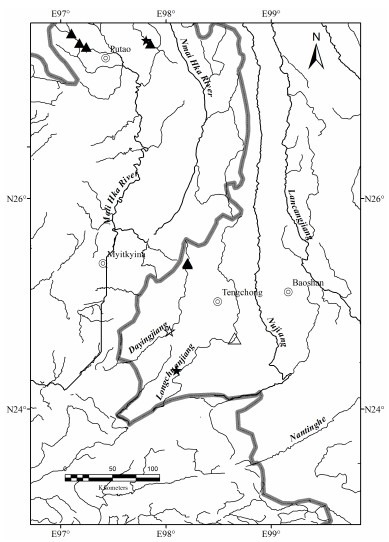
Distribution of *Tor yingjiangensis* (☆: type locality; ★: non-type locality); and *Tor qiaojiensis* (△: type locality; ▲: non-type locality)

**Distribution**: *Tor yingjiangensis* is usually found in the main stem of the Mali Hka River, and sought fresh from the Putao and Myitkyina fish markets in the Irrawaddy Basin of northern Myanmar, and in the Longchuanjiang and Dayingjiang rivers of Yunnan, China (Figure 7).

**Remarks**: Myanmar specimens well-match *Tor yingjiangensis* from the Dayingjiang River of Yunnan, China, except for some minor differences in counts and measurements: D-LL 3.5–4 (vs. 4–4.5), V-LL 3 (vs. 3–3.5), circumpeduncular scales 10–12 (vs. 12), caudal-peduncle length 13.4%–15.8% SL (vs. 11.3–14.8), and caudal-peduncle depth 10.7%–12.7% SL (vs. 11.1–13.3).

### *Tor qiaojiensis* Wu & Yao in Wu, 1977

*Tor* (*Tor*) *qiaojiensis* Wu & Yao, in Wu, 1977: 159–160 (Qiaojie, Yunnan, China); Chu & Cui in Chu & Chen, 1989 (Lianghe, Yingjiang, Tengchong, Yunnan, China).

**Material examined**: *Tor qiaojiensis*, KIZ2014005930–931, 2 ex., 46.1–53.7 mm SL, stream near Rat Baw Village, Naung Mun Township, Putao District, Kachin State, Myanmar (N27°28'20.89", E97°51'06.86", 558 m a.s.l.), collected by Xiao-Yong Chen and Tao Qin, 2 December 2014; KIZ2015006380–381, SEABRI20150277–281, SEABRI20150830–837, 15 ex., 62.8–189.7 mm SL, Zeyar Stream, Irrawaddy Basin, Zeyar Dan Village, Putao District, Kachin State, Myanmar (N27°34'12.08", E97°06'02.73", 1 036 m a.s.l.), collected by Xiao-Yong Chen, Tao Qin and Shu-Sen Shu, 9 December 2015; SEABRI20150459–462, 4 ex., 58.2–62.8 mm SL, Nam Ru Stream, Irrawaddy Basin, War Sar Dan Village, Putao District, Kachin State, Myanmar (N27°28'43.14", E97°10'43.23", 830 m a.s.l.), collected by Xiao-Yong Chen, Tao Qin, and Shu-Sen Shu, 17 December 2015; SEABRI20150464–466, 3 ex., 54.1–126.8 mm SL, Patheik Stream, Irrawaddy Basin, Putao District, Kachin State, Myanmar (N27°26'27.51", E97°14'30.33", 790 m a.s.l.), collected by Xiao-Yong Chen, Tao Qin, and Shu-Sen Shu, 19 December 2015.

**Comparative material examined**: KIZ2006012423–455, 39.9–104.8 mm SL, 33 ex., Guyong River (tributary of Binglangjiang River, upper Irrawaddy River), Guyong Town, Tengchong County, Yunnan, China; KIZ2006012418–422, 86.4–145.5 mm SL, 5 ex., Binglangjiang River (upper Dayingjiang River, Irrawaddy Basin), Guyong Town, Tengchong County, Yunnan, China.

**Figure 5:**
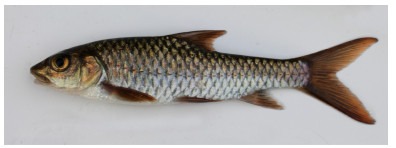
Lateral view of *Tor qiaojiensis* before preservation, SEABRI20150837, 189.7 mm SL

**Figure 6:**
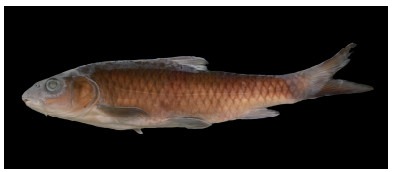
Lateral view of *Tor qiaojiensis* after preservation in formalin, SEABRI20150837, 189.7 mm SL

**Description**: Based on the literature (Chu & Chen, 1989; Wu, 1977), this species can be distinguished from other congeners of *Tor* by the unique combination of the following features: body elongate and head compressed, dorsal profile gradually increasing, reaching apex just in front of dorsal-fin origin, head length slightly longer or equal to maximum body depth, anus close to anal-fin origin, caudal deeply forked (Figures 5, 6). Mouth subterminal, snout blunt, lips fleshy, median lobe of lower lip undeveloped, two pairs of barbels, rostral barbels longer than maxillary barbels (Figure 4A). Dorsal-fin with four unbranched and nine branched rays, last unbranched ray osseous and only articulated on distal tip. Lateral line complete, lateral-line scales 29–30, D-LL 4.5, V-LL 2.5–3, circumpeduncular scales 10.

Counts and morphometric measurements of examined specimens for mahseers are listed in Table 1.

**Color in life**: Body and head dark grey on back, golden on lateral side, white on ventral side. Golden on opercule and lighter on preopercule. All fins dark brown. Scales form dark network on flank (Figure 5).

**Color in preservative**: Body and head dark grey on back, brown on lateral side, white on ventral side. All fins dark grey. Scales form dark network on flank (Figure 6).

**Distribution**: Found from mountain tributaries of the Mali Hka River Basin in the Rat Baw, Zeyar Dan, War Sar Dan villages, Putao, Myanmar. In China, this species is mainly distributed in tributaries of the Longchuanjiang River and Dayingjiang River in the upper Irrawaddy Basin (Chu & Chen, 1989; Wu, 1977) (Figure 7).

**Remarks**: Myanmar specimens well-match *Tor qiaojiensis* from Yunnan, China, except for some minor differences in counts and measurements: D-LL 4.5 (vs. 4.5–5.5), circumpeduncular scales 10 (vs. 10–12), head length 26.3%–31.4% SL (vs. 27.7–30.6), body depth 24.7%–28.1% SL (vs. 27.3–31.4), caudal-peduncle length 15.5%–17.4% SL (vs. 13.9–18.8), and caudal-peduncle depth 11.1%–12.5% SL (vs. 9.7–11).

### *Garra qiaojiensis* Wu & Yao in Wu, 1977

*Garra qiaojiensis* Wu & Yao, in Wu, 1977: 238-239 (Qiaojie, Yunnan, China); Chu & Cui, in Chu & Chen, 1989 (Tuantian and Gudong of Tengchong, Lianghe, Husa of Longchuan, Yunnan, China).

**Material examined**: SEABRI20140185–193, 9 ex., 76.2–94.9 mm SL, main stem of Mali Hka River, Wurunga Village, Naung Mun Township, Putao District, Kachin State, Myanmar (N27°30'15.3", E97°48'48.2", 543 m a.s.l.), collected by Xiao-Yong Chen and Tao Qin, 30 November to 7 December 2014; SEABRI20150231–234, SEABRI20150414, 5 ex., 118.8–165.9 mm SL, Zeyar Stream, Mali Hka Basin, Zeyar Dan Village, Putao District, Kachin State, Myanmar (N27°34'12.08", E97°06'02.73", 1 036 m a.s.l.), collected by Xiao-Yong Chen, Tao Qin, and Shu-Sen Shu, 9 & 14 December 2015; KIZ2016007376-377, SEABRI20160188-190, 5 ex., 60.7–73.4 mm SL, from Putao fish market, Putao District, Kachin State, Myanmar, collected by Xiao-Yong Chen, Tao Qin, and Shu-Sen Shu, 6 August 2016.

**Comparative material examined**: KIZ2005002680–694, 49.4–72.1 mm SL, 15 ex., Zhina River (tributary of Dayingjiang River), Zhina Town, Yingjiang County, Yunnan, China; KIZ2006011157, 92.9 mm SL, Binglangjiang River (upper Dayingjiang River), Guyong Town, Tengchong County, Yunnan, China; KIZ2006011169–174, 81.1–118.8 mm SL, 6 ex., Longchuanjiang River, Wuhe Town, Tengchong County, Yunnan, China; KIZ2006004475–476, 68.7–75.9 mm SL, 2 ex., Minglang River (tributary of Longchuanjiang River), Hehua Town, Tengchong County, Yunnan, China.

**Figure 8:**
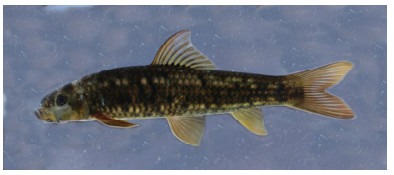
Lateral view of *Garra qiaojiensis* in life, SEABRI20140185, 76.2 mm SL

**Figure 9:**
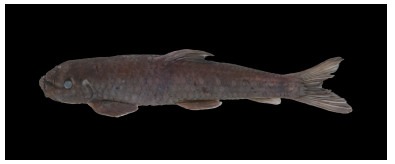
Lateral view of *Garra qiaojiensis* after preservation in formalin, SEABRI20140185, 76.2 mm SL

**Figure 10:**
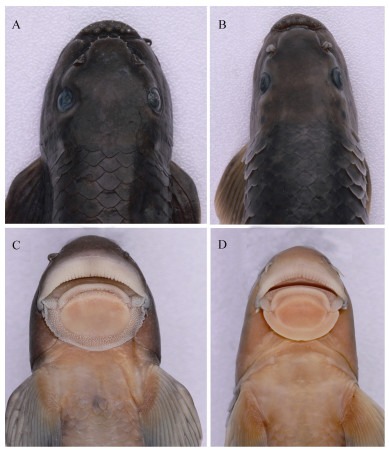
Dorsal (top) and ventral (bottom) view of head

**Description**: Specimens examined were identified as *Garra qiaojiensis* according to the following characters (Chu & Chen, 1989; Wu, 1977): Body rounded and caudal peduncle compressed (Figures 8, 9). Head rounded and moderately large, head wider than deep, eyes small, mouth inferior, snout moderately rounded, two pairs of short barbels, mental disc elliptical (Figure 10A, C). Dorsal fin with four simple and eight branched rays. Lateral line complete, with 34–35 scales, D-LL 4, V-LL 2.5–3, circumpeduncular scales 12, predorsal scales 10–11.

Counts and morphometric measurements of examined specimens are listed in Table 2.

**Table 2:** Comparisons of counts and proportional measurements of *G*. *qiaojiensis* and *G*. *bispinosa*

	***G*. *qiaojiensis* (*n*=19)**	***G*. *qiaojiensis*^1^(*n*=13)**	***G*. *bispinosa* (*n*=42)**	***G*. *bispinosa*^2^(*n*=27)**
Total length (mm)	80.5–165.9	61.5–160.0	65.5–150.2	N/A
Standard length (mm)	60.7–138.1	49.0–124.0	49.2–118.9	75.7–127.8
Dorsal-fin rays	ⅳ, 8	ⅳ, 8	ⅳ, 8	ⅳ, 7–8
Anal-fin rays	ⅲ, 5	ⅲ, 5	ⅲ, 5	ⅲ, 5
Pectoral-fin rays	ⅰ, 13	ⅰ, 13–15	ⅰ, 13	ⅰ, 13–14
Pelvic-fin rays	ⅰ, 7–8	ⅰ, 8	ⅰ, 7	ⅰ, 7–8
Lateral-line scales	34–35	33–36	34–35	34–35
D-LL	4	3–4	4	4
V-LL	2.5–3	2.5–3	2.5–3	2.5–3
Predorsal scales	10–11	9–11	10–11	9–11
Circumpeduncular scales	12	12	16	16
**Percentage of SL (%)**				
Body depth	20.7–23.9	20.5–23.3	20.0–25.4	20.8–24.1
Head length	22.4–26.2	21.7–25.9	22.6–25.1	22.6–24.6
Head depth	15.4–18.0	15.9–18.2	16.7–18.2	15.4–17.9
Head width	17.3–20.5	17.8–19.0	17.0–19.5	16.4–19.3
Caudal-peduncle length	12.4–15.7	12.3–15.8	13.9–16.3	14.7–18.5
Caudal-peduncle depth	11.5–13.2	11.1–13.3	11.6–13.8	12.4–13.6
**Percentage of HL (%)**				
Snout length	37.1–50.8	39.3–50.4	42.6–50.4	49.5–56.5
Eye diameter	19.6–25.0	19.1–25.6	18.1–24.4	17.9–21.7
^1^: from Chu & Chen (1989). Some data were converted for comparison; ^2^: from Zhang (2005).				

**Color in life**: Head and body dark olive green mottled with yellow, ventral side of body white. Dark spot at upper extremity of gill opening. All fins orange, rays dark, membrane light (Figure 8).

**Color in preservative**: Body dark brown on dorsal and lateral sides, brown on ventral side. All fins brown, rays dark (Figure 9).

**Figure 14:**
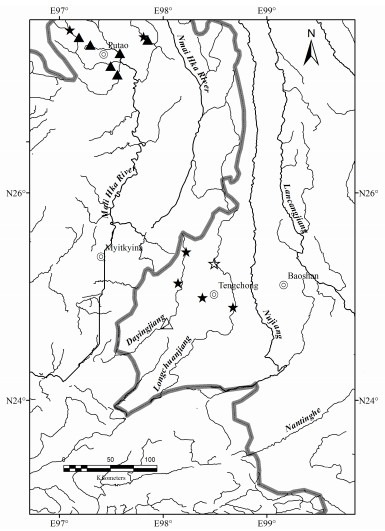
Distribution of *Garra qiaojiensis* (☆: type locality; ★: non-type locality) and *Garra bispinosa* (△: type locality; ▲: non-type locality)

**Distribution**: In China, *Garra qiaojiensis* is currently known from the Dayingjiang River and Longchuanjiang River of Yunnan (Chu & Chen, 1989; Wu, 1977). In Myanmar, it occurs in streams and tributaries of the Mali Hka River Basin around Putao (Figure 14).

### *Garra bispinosa* Zhang, 2005

*Garra bispinosa* Zhang, 2005: 9–15 (Yingjiang, Yunnan, China).

*Garra orientalis*: Chu & Cui, in Chu & Chen, 1989 (in part, Nabang of Yingjiang, Luxi, Wanting, Yunnan, China).

**Materials examined**: SEABRI20140104, 1 ex., 112.2 mm SL, stream near Rat Baw Village, Naung Mun Township, Putao District, Kachin State, Myanmar (N27°28'20.89", E97°51'06.86", 558 m a.s.l.), collected by Xiao-Yong Chen and Tao Qin, 2 December 2014; SEABRI20150036–037, 2 ex., 79.6–96.5 mm SL, stream near Upper Chan Khaung Village, Putao District, Kachin State, Myanmar (N27°25'25.33", E97°17'51.33", 446 m a.s.l.), collected by Xiao-Yong Chen, Tao Qin, and Shu-Sen Shu, 5 December 2015; SEABRI20150125–148, 23 ex., 69.6–95.3 mm SL (Table 2), Monlar Stream, War Sar Dan Village, Putao District, Kachin State, Myanmar (N27°29'49.38", E97°11'20.31", 839 m a.s.l.), collected by Xiao-Yong Chen, Tao Qin, and Shu-Sen Shu, 7 December 2015; SEABRI20151251-259, 9 ex., 86.7–117 mm SL, A Dae Htar Thai Stream, Putao District, Kachin State, Myanmar (N27°13'18.57", E97°29'41.73", 426 m a.s.l.), collected by Xiao-Yong Chen, Tao Qin, and Shu-Sen Shu, 23 December 2015; SEABRI20151112, 1 ex., 118.9 mm SL, Tanjar Stream near Lone Shar Yan Village, Putao District, Kachin State, Myanmar (N27°08'18.92", E97°33'34.38", 422 m a.s.l.), collected by Xiao-Yong Chen, Tao Qin, and Shu-Sen Shu, 26 December 2015; SEABRI20151359-364, 6 ex., 51.4–89.6 mm SL, Nan Pat Stream, Machanbaw Township, Putao District, Kachin State, Myanmar (N27°20'41.21", E97°34'57.75", 405 m a.s.l.), collected by Xiao-Yong Chen, Tao Qin, and Shu-Sen Shu, 29 December 2015.

**Figure 11:**
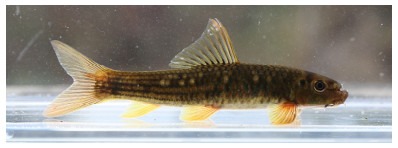
Lateral view of *Garra bispinosa* in life, SEABRI20150470, 74.9 mm SL

**Figure 12:**
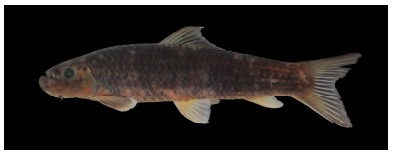
Lateral view of *Garra bispinosa* after preservation in formalin, SEABRI20151112, 145.5 mm SL

**Description**: Examined specimens well-matched *Garra bispinosa* (Zhang, 2005), and can be distinguished from congeners by the following characters: Body elongate, dorsal and ventral profile slightly convex (Figures 11, 12). Head moderately large and slightly compressed, longer than deep, deep less than wide, eyes small, two pairs of barbels shorter than eye diameter, maxillary barbels shorter than rostral ones, mental disc elliptical and shorter than wide, conspicuous proboscis with tubercles, anteriorly bilobed in large specimens, snout slightly pointed and tip with conspicuous groove (Figure 10B, D). Lateral line complete, with 34–35 scales, circumpeduncular scales 16. Dorsal fin with four simple and eight branched rays, D-LL 4, V-LL 2.5–3, predorsal scales 10–11.

Counts and morphometric measurements of examined specimens are listed in Table 2.

**Color in life**: Body and head dark olive green. Ventral side of body white. Body with six dark stripes on lateral side, more prominent posterior of dorsal-fin base. All fins orange, rays dark (Figure 11).

**Color in preservative**: Body dark brown on dorsal and lateral sides, brown on ventral side. Dark spot at upper extremity of gill opening. All fins yellowish, rays dark (Figure 12).

**Figure 13:**
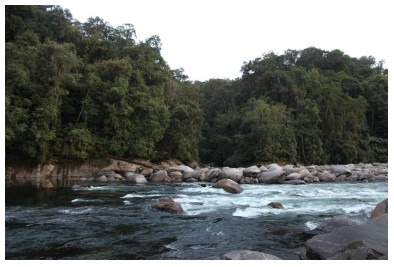
Tributary of the Mali Hka River at Wurunga Village, Naung Mun Township, Putao District, Kachin State, Myanmar

**Distribution**: In China, *Garra bispinosa* is currently known from Dayingjiang River and Longchuanjiang River, upper Irrawaddy River in Yunnan (Chen, 2013; Zhang, 2005). In Myanmar, it occurs in streams and tributaries of the Mali Hka River Basin around Putao, northern Myanmar (Figures 13, 14).

### *Schizothorax oligolepis* Huang, 1985

*Schizothorax oligolepis* Huang, 1985: 209-210 (Tongbiguan of Yingjiang, Yunnan, China); Yang et al., 2013: 365-366 (Dayingjiang, Yunnan, China).

*Schizothorax* (*Schizothorax*) *oligolepis*: Mo, in Chu & Chen, 1989 (Tongbiguan of Yingjiang, Yunnan, China).

**Material examined**: KIZ2015006382–383, SEABRI20150220–225, 283–291, 17 ex., 61.2–91.6 mm SL, Zeyar Stream, Irrawaddy Basin, Zeyar Dan Village, Putao District, Kachin State, Myanmar (N27°34'12.08", E97°06'02.73", 1 036 m a.s.l.), collected by Xiao-Yong Chen, Tao Qin, and Shu-Sen Shu, 9 December 2015; SEABRI20150347, 350–351, 355, 362, 364, 375–377, 9 ex., 66.4–152.1 mm SL, upper tributary of Ponyin Stream, Zeyar Dan Village, Putao District, Kachin State, Myanmar (N27°35'43.87", E96°59'46.33", 1 500 m a.s.l.), collected by Tao Qin and Shu-Sen Shu, 11 & 12 December 2015; SEABRI20150408–413, 6 ex., 72.6–84.4 mm SL, Ponyin Stream, Zeyar Dan Village, Putao District, Kachin State, Myanmar (N27°33'51.77", E97°05'25.11", 1 072 m a.s.l.), collected by Xiao-Yong Chen, Tao Qin, and Shu-Sen Shu, 14 December 2015; SEABRI20150497–507, 11 ex., 57–100.5 mm SL, Zan Shaw Stream, War Sar Dan Village, Putao District, Kachin State, Myanmar (N27°28'00.26", E97°13'05.73", 1 134 m a.s.l.), collected by Xiao-Yong Chen, Tao Qin, and Shu-Sen Shu, 18 December 2015.

**Comparative material examined**: KIZ2016002803, 905–913, 920, 11 ex., 48.5–132.3 mm SL, Erganya River (tributary of Dayingjiang), Tongbiguan Town, Yingjiang County, Yunnan, China; KIZ2014005075-082, 62.3–91.6 mm SL, 8 ex., Dazhupeng River (tributary of Dayingjiang River), Zhina Town, Yingjiang County, Yunnan, China; KIZ2014004705–709, 48.8–71.6 mm SL, 5 ex., Zhongling River (tributary of Dayingjiang River), Zhina Town, Yingjiang County, Yunnan, China.

**Figure 15:**
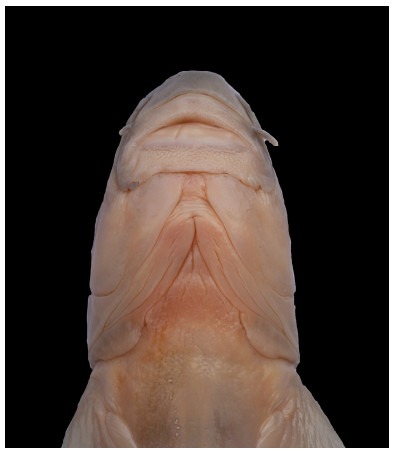
Ventral view of head, *Schizothorax oligolepis*, SEABRI20150221, 71.1 mm SL

**Figure 16:**
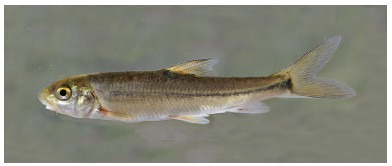
Lateral view of *Schizothorax oligolepis* in life, SEABRI20150221, 71.1 mm SL

**Figure 17:**
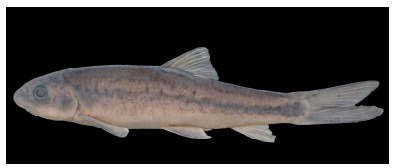
Lateral view of *Schizothorax oligolepis* after preservation in formalin, SEABRI20150221, 71.1 mm SL

**Description**: Combined with the diagnostic characters from the original description, the examined specimens can be distinguished as *Schizothorax oligolepis* based on the following characters: Body moderately elongate, head and caudal peduncle compressed, dorsal profile arched more than ventral (Figures 16, 17). Snout blunt and rounded, lower jaw with sharp horny sheath, lower lip well-developed, papillated, postlabial groove continuous (Figure 15). Lateral line complete and slightly straight, scales minute, obvious scales present on thorax and abdomen. Dorsal-fin with four simple and 7–8 branched rays, last unbranched dorsal fin osseous and serrated on lower part, soft on distal tip, dorsal-fin origin anterior of pelvic-fin origin, dorsum with speckles.

Counts and partial comparative measurements of examined materials are presented in Table 3.

**Table 3:** Counts and proportional measurements of *Schizothorax oligolepis*.

	***S. oligolepis* (*n*=43)**	***S. oligolepis*^1^(*n*=21)**
Total length (mm)	75.3–193.7	N/A
Standard length (mm)	57.0–152.1	110.0–176.0
Dorsal-fin rays	Ⅳ, 7–8	Ⅳ, 7
Anal-fin rays	ⅲ, 4–5	ⅲ, 5
Pectoral-fin rays	ⅰ, 16–18	ⅰ, 17–18
Pelvic-fin rays	ⅰ, 8–9	ⅰ, 9–10
**Percentage of SL (%)**		
Body depth	21.3–26.4	20.9–27.3
Head length	22.7–27.5	21.8–27.1
Head depth	15–19.4	16.2–18.7
Head width	14–16.5	14.8–17
Caudal-peduncle length	15.1–17.7	15.3–18.3
Caudal-peduncle depth	9.6–11.2	10.1–12.8
**Percentage of HL (%)**		
Snout length	28.6–34.2	29.1–33.9
Eye diameter	22.5–29.3	23.7–28.9
^1^: from Huang (1985). Some data were converted for comparison.		

**Color in life**: Head and body olive green, dorsum dark, with speckles, ventral side silver. Black longitudinal stripe above and along lateral line present. Semicircular black blotch on caudal-fin base. Eyes reddish on dorsal part. Anal fins hyaline, other fins light orange, dorsal and caudal fins with greyish margin (Figure 16).

**Color in preservative**: Head and body brown, dorsum dark, with speckles, ventral side light. Black longitudinal stripe above and along lateral line present. Semicircular black blotch on caudal-fin base. Fin rays grey (Figure 17).

**Figure 18:**
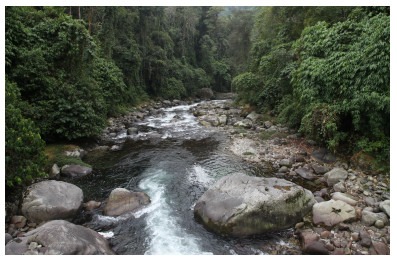
Ponyin Stream, a tributary of Mali Hka River close to Zeyar Dan Village, Putao District, Kachin State, Myanmar

**Figure 19:**
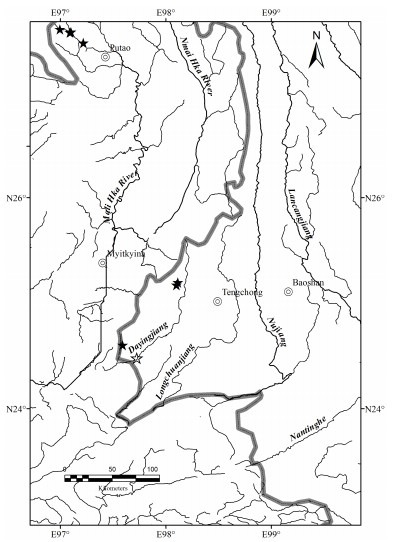
Distribution of *Schizothorax oligolepis* (☆: type locality; ★: non-type locality)

**Distribution**: It occurs in mountain streams in the Hponkanrazi Wildlife Sanctuary, including Zeyar Stream and Ponyin Stream of the upper Mali Hka River around Putao, and upper tributaries of Dayingjiang River, Yunnan, China (Chu & Chen, 1989; Huang, 1985) (Figures 18, 19).

**Remarks**: The last unbranched dorsal fin is osseous and serrated on the lower part, but soft on distal tip in the Myanmar specimen, which is weaker in the Chinese specimens.

## ACKNOWLEDGEMENTS

We sincerely thank Shu-Sen Shu from the Kunming Institute of Zoology, Chinese Academy of Sciences (CAS), Yun-Hong Tan and Bin Yang from the Xishuangbanna Tropical Botanical Garden (XTBG), CAS, and Shwe Lone and Kyi Kyi Khaing from the Forest Research Institute, Forest Department, Ministry of Environmental Conservation and Forestry, Myanmar, for their contribution to the field survey. We especially thank Rui-Chang Quan and Ren Li from XTBG, CAS, for their heartfelt support in the field.
